# Continuous infusion versus intermittent infusion of vancomycin in critically ill patients undergoing continuous venovenous hemofiltration: a prospective interventional study

**DOI:** 10.1186/s12879-022-07618-6

**Published:** 2022-08-02

**Authors:** Jinhui Xu, Lufen Duan, Jiahui Li, Fang Chen, Xiaowen Xu, Jian Lu, Zhiwei Zhuang, Yifei Cao, Yunlong Yuan, Xin Liu, Jiantong Sun, Qin Zhou, Lu Shi, Lian Tang

**Affiliations:** 1 Department of Pharmacy, The Affiliated Suzhou Hospital of Nanjing Medical University, Suzhou Municipal Hospital, Suzhou, 215002 China; 2 Emergent Intensive Care Unit, The Affiliated Suzhou Hospital of Nanjing Medical University, Suzhou Municipal Hospital, Suzhou, 215002 China; 3 Intensive Care Unit, The Affiliated Suzhou Hospital of Nanjing Medical University, Suzhou Municipal Hospital, Suzhou, 215002 China; 4 Medical Laboratory, The Affiliated Suzhou Hospital of Nanjing Medical University, Suzhou Municipal Hospital, Suzhou, 215002 China

**Keywords:** Vancomycin, Continuous venovenous hemofiltration, Continuous infusion, Intermittent infusion, Prospective interventional study

## Abstract

**Background:**

A prospective interventional study comparing outcomes in critically ill patients receiving intermittent infusion (II) or continuous infusion (CI) of vancomycin during continuous venovenous hemofiltration (CVVH) is lacking. The objective of this study was to compare the pharmacokinetic/pharmacodynamics (PK/PD) target attainment, therapeutic efficacy and safety among critically ill patients who received CI or II of vancomycin in a prospective interventional trial and to explore the correlations of effluent flow rate (EFR) with PK/PD indices.

**Methods:**

This prospective interventional study was conducted in two independent intensive care units (ICUs) from February 2021 to January 2022. Patients in one ICU were assigned to receive CI (intervention group) of vancomycin, whereas patients in the other ICU were assigned to receive II regimen (control group). The primary outcome was to compare the PK/PD target attainment, including target concentration and target area under the curve over 24 h to minimum inhibitory concentration (AUC_24_/MIC).

**Results:**

Overall target attainment of PK/PD indices was higher with CI compared with II, irrespective of target concentration (78.7% vs. 40.5%; P < 0.05) or AUC_24_/MIC (53.2% vs. 28.6%; P < 0.05). There were no significant differences in clinical success (72.2% vs. 50.0%; P = 0.183) and microbiological success (83.3% vs. 75.0%, P = 0.681) between the patients treated with CI or II of vancomycin. Adverse reactions occurred at similar rates (0.0% vs. 4.4%; P = 0.462), and mortality between the two modalities was also not significant different (21.7% vs. 17.9%; P = 0.728). Correlation analysis showed a weak to moderately inverse correlation of EFR with observed concentration (r = − 0.3921, P = 0.01) and AUC_24_/MIC (r = − 0.3811, P = 0.013) in the II group, whereas the correlation between EFR and observed concentration (r = − 0.5711, P < 0.001) or AUC_24_/MIC (r = − 0.5458, P < 0.001) in the CI group was stronger.

**Conclusion:**

As compared to II, CI of vancomycin in critically ill patients undergoing CVVH was associated with improved attainment of PK/PD indices. Furthermore, the inverse correlation of PK/PD indices with EFR was stronger among patients treated with CI of vancomycin.

*Trial registration* The trial was registered in the Chinese clinical trial registration center (21/01/2021-No. ChiCTR2100042393).

## Background

Infections caused by multidrug-resistant Gram-positive pathogens persists a major public health crisis among critically ill patients, leading to increased morbidity and mortality [1]. Vancomycin remains the first-line therapy against these pathogens, including methicillin-resistant *Staphylococcus aureus* (MRSA), methicillin resistant coagulase negative staphylococci (MRCNS) [2]. Appropriate dosing of vancomycin in critically ill patients is high challenging due to large inter- and intra-individual pharmacokinetic variability [3], including distinct volume distribution and clearance [4]. Moreover, continuous kidney replacement therapy (CKRT) is commonly utilized in critically ill patients with acute kidney failure, especially for those hemodynamically unstable subjects [5]. The pharmacokinetic/pharmacodynamics (PK/PD) parameters of vancomycin may be further influenced by the type of CKRT and parameter settings [5], hence complicating dosing strategies.

Therefore, therapeutic drug monitoring (TDM) is recommended to be employed during vancomycin therapy [6], in order to maximize its efficacy, while minimizing the likelihood of toxicity. In critically ill patients undergoing CKRT, standard conventional intermittent infusion (II) may not guarantee achievement of target PK/PD indices, as reported by Omrani et al. [7], only 34.3% patients maintained the desired target range. Consequently, there’s growing interest in employing continuous infusion (CI) schedule to increase the probability of target attainment.

Dosing targets for steady-state concentration (C_ss_) and trough concentration (C_min_) varied in the previous studies [8–10], but the most common target C_ss_ was 15–25 mg/L for CI and a C_min_ of 15–20 mg/L for II. A meta-analysis found that CI of vancomycin in critically ill patients was associated with a 53% reduction in the odds of developing acute kidney injury (AKI) and 2.63-fold greater odds of pharmacokinetic target attainment in comparison with II regimen [11]. Nevertheless, it analyzed a diverse patient population derived from a variety of studies, which may cause bias toward finding significant results. As compared to II, critically ill patients during continuous venovenous hemofiltration (CVVH) administrated with CI of vancomycin achieved the target concentration faster and consistently kept the target level [12]. However, the investigation was retrospective, and intrinsic limitations associated with retrospective studies, including selection biases and heterogeneity in dosing strategies, might influence the outcomes. The Infectious Disease Society of America (IDSA) [13] and the Chinese Pharmacological Society (CPS) [14] have recently updated its guidelines about TDM of vancomycin, target C_ss_ is 15–25 mg/L for CI and a C_min_ of 10–20 mg/L for II is recommended for patients with multidrug-resistant Gram-positive infections. Furthermore, target area under the curve (AUC)-based TDM has been proposed as an alternative approach due to its reduced risk of nephrotoxicity and a target area under the curve over 24 h to minimum inhibitory concentration (AUC_24_/MIC) ratio of 400–650 has always been advocated in the updated guidelines. To date, a prospective interventional study comparing target attainment of PK/PD indices recommended by the updated guidelines between the two dose modalities in critically ill patients on CVVH is lacking.

The aim of this study was to compare, in a prospective interventional trial, the target attainment of trough concentration (C_min_), steady-state concentration (C_ss_) and target area under the curve over 24 h to minimum inhibitory concentration (AUC_24_/MIC), therapeutic efficacy and safety in critically ill patients on CVVH receiving vancomycin via either II or CI strategy. The correlations of EFR with PK/PD indices in critically ill patients receiving vancomycin via the two modalities during CVVH were also analyzed.

## Methods

### Setting

This was a prospective non-randomized interventional study that was conducted in two independent ICUs of the Affiliated Suzhou Hospital of Nanjing Medical University from February 2021 to January 2022. All study procedures were approved by the local ethics committee (Approval No. K-2020-011-K01), and registered in the Chinese clinical trial registration center (registration number: ChiCTR2100042393) prior to enrollment of patients. Written informed consent was obtained from the participant or a surrogate decision maker before any study-specific procedures.

### Study population

The inclusion criteria were as follows: (1) empirical or target treatment with vancomycin; (2) AKI requiring CVVH; (3) anuria (24-h urine volume < 100 mL) [15] during CVVH; (4) age ≥ 18 years old. Patients were excluded if they met any of the following criteria: (1) concomitantly undergoing extracorporeal membrane oxygenation (ECMO); (2) the CVVH mode changed to other modes of CKRT or intermittent kidney replacement treatment (IRRT) during vancomycin treatment; (3) receiving the first dose of vancomycin six or more hours prior to the initiation of CVVH; (4) incomplete or missing demographic and clinical information.

### Continuous kidney replacement therapy settings

CVVH was the mode utilized in all patients. CVVH was performed using a PrismaFlex® apparatus (Gambro Hospal, Bologna, Italy) with polymethyl methacrylate membrane (Hemofeel CH-1.0N; Toray Medical, Tokyo, Japan) haemofilters. Vascular access was achieved through a double-lumen catheter inserted into the femoral vein. Anticoagulation was achieved by means of either heparin or citrate within the CKRT circuit. Blood flow was set at around 180 to 200 mL/min, flow rates of pre-dilution and post-dilution replacement fluid ranged from 2000 to 3000 mL/h.

### Data collection

The following demographic and clinical data were collected for each patient, including age, gender, height, weight, acute physiology and chronic health evaluation II (APACHE II) score, sequential organ failure assessment (SOFA) score, comorbidities, complications, the type of infection, microbiological isolates and MIC (if possible), 28-day mortality. Laboratory values, such as alanine aminotransferase (ALT), albumin, serum creatinine at baseline and on the last day of vancomycin administration were also recorded. Treatment regimen including dose, intervals, duration of vancomycin administration and concomitant antibiotic therapy were collected. CVVH settings, including blood flow rate, pre-dilution replacement fluid flow rate, post-dilution replacement fluid flow rate, net removal fluid rate, haemofiltration rate and effluent flow rate (EFR) were gathered.

### Dosing regimen

Patients in one ICU were assigned to receive CI (intervention group) of vancomycin, whereas patients in the other ICU were assigned to receive II regimen (control group). The conventional II regimen was based on the guidelines as follows [13]: (1) a loading dose of 20–25 mg/kg followed by a maintenance dose of 7.5–10 mg/kg q12h for patients on CVVH with EFR of 20–25 mL/kg/h; (2) empirically increasing dose to 10–13 mg/kg q12h for those undergoing CVVH with EFR at 25–40 mL/kg/h, and 13–15 mg/kg q12h for EFR more than 40 mL/kg/h. Maintenance dose was adjusted during therapy on the basis of trough concentration. Patients in the CI group were given a 20–25 mg/kg loading dose followed immediately by maintenance with CI of vancomycin [13, 16]. The initial maintenance dose was estimated based on Eq. 1 [16]:

1 $$Dosage\,via\, C\,I\left( {{\text{mg}}/24{\text{h}}} \right)\, = \,\frac{{CVVH\, intensity\,\left( {mL/kg/h} \right) - 0.392}}{8.368}\, \times \,20\left( {mg/L} \right)\, \times \,24\left( h \right)$$

where 20 mg/L is the desired C_ss_ concentration, CVVH intensity is generally expressed in terms of EFR (mL/kg/h) [16, 17].

The subsequent dosing regimens were adjusted as follows: (1) therapy of vancomycin was temporarily terminated if CVVH circuit stopped or clotted off; resumed infusion when CVVH circuit restarted; (2) if the C_ss_ was less than 15 mg/L, the infusion dose was increased by 25%; (3) if the C_ss_ exceeded 25 mg/L, the CI was suspended for 4 h and daily dose was reduced by 50% or 25% in the case of concentrations above 35 mg/L or within the range of 25–35 mg/L, respectively.

### Blood and ultrafiltrate sampling and assay of vancomycin concentration

Trough concentration blood samples were collected 24 h after the treatment initiation, and 30 min prior to the next dose of vancomycin for patients with II, while for CI regimen, blood samples were obtained 24 h after the commencement of the vancomycin infusion schedule. Ultrafiltrate samples were collected at the same time points. Vancomycin concentrations in serum and ultrafiltrate samples were quantified via a chemiluminescence enzyme immunoassay using Architect i2000SR analyzer with AxSYM vancomycin assay (Abbott, Illinois, USA).

### Primary outcome

The primary outcome of the study was to compare attainment of target PK/PD variables, including C_min_ (II group), C_ss_ (CI group) and AUC_24_/MIC.

For patients receiving CI, the AUC_24_ was calculated using the steady-state concentrations multiplied by 24 h. AUC_24_ during II was calculated as [18]:

2 $${\text{AUC}}_{24} = \frac{Daily\,dose}{{CL_{vanc} }}$$

The total drug clearance (CL_total_) was calculated from the sum of patient’s clearance (non-renal; CL_NR_ and renal clearance; CL_R_) and CRRT extracorporeal clearance (CL_CRRT_) using the following equation:

3 $$CL_{total} = CL_{NR} + CL_{R} + CL_{CVVH}$$

We included patients with anuria during CVVH. Most AKI patients have no urine output; therefore, the renal clearance of these patients was assumed as 0 mL/min. CL_total_ was calculated using pharmacokinetic software Phoenix NLME (version 8.3; Certara USA, Inc., Princeton, NJ, USA).

The vancomycin clearance of CVVH (CL_CVVH_) is calculated for each participant according to Eqs. 4–7 [19, 20]:

4 $$S_{C} = \frac{{C_{UF} }}{{C_{SE} }}$$

5 $$CL_{{CVVH\left( {post} \right)}} = Q_{f} \times SC$$

6 $$CL_{{CVVH\left( {pre} \right)}} = Q_{f} \times SC \times \frac{{Q_{b} }}{{Q_{b} + Q_{spre} }}$$

7 $$CL_{CVVH} = CL_{{CVVH\left( {post} \right)}} + CL_{{CVVH\left( {pre} \right)}}$$

where S_C_ is sieving coefficient, C_SE_ is the serum vancomycin concentration in the ultrafiltrate collection period (mg/L) and C_UF_ is the concentration of vancomycin in the ultrafiltrate (mg/L), CL_CVVH (post)_ and CL_CVVH (pre)_ are hemofilter clearance (L/h) for CVVH using post-filter hemodilution and pre-filter hemodilution, respectively, Q_f_ is the haemofiltration rate (L/h), Q_b_ is the blood flow rate (L/h) and Q_spre_ is the pre-dilution replacement fluid flow rate (L/h).

The AUC_24_/MIC ratio was obtained by dividing the AUC_24_ by MIC of microbiological isolates, assuming a vancomycin MIC of 1 mg/L for empirical treatment. Target C_ss_ was 15–25 mg/L for CI group and a C_min_ of 10–20 mg/L for II group was recommended for patients with multidrug-resistant Gram-positive infections [13, 14]. When transitioning to AUC_24_/MIC monitoring, the AUC_24_/MIC target has always been advocated as 400 to 650 for vancomycin [14]. Variations in serum concentrations and AUC_24_/MIC, expressed as relative standard deviation (RSD), were calculated using the formula: RSD = 100 × standard deviation (SD)/mean.

### Secondary outcomes

Secondary outcomes included clinical efficacy, microbiological efficacy, 28-day mortality, adverse reaction and the correlations of EFR, daily dose with PK/PD indices. Clinical efficacy and microbiological efficacy were evaluated in patients with an identified Gram-positive pathogenic organism, whereas 28-day mortality and adverse reactions were assessed for all the patients. Clinical outcome was evaluated as treatment success or failure at the end of vancomycin therapy [21]. Clinical success was defined as resolution or improvement of clinical signs and symptoms attributable to infection, and clinical failure was defined as persistence or progression of clinical signs and symptoms during vancomycin therapy. Bacterial eradication was assessed as documented eradication, presumed eradication, documented persistence, presumed persistence. Microbiological success was defined as documented eradication or presumed eradication of Gram-positive bacteria. According to criteria for causality assessment of adverse reaction [22], cases that judged to be possible, probable, or certain were recognized as related to vancomycin treatment. Leukopenia was defined as a leukocyte count less than 4 × 10^9^/L [23]. To assess the association of PK/PD variables with daily dose or EFR, correlative analyses was used.

### Statistical analysis

According to the published literature, the rates of concentrations in the target range were 100% and 19% in CI and II regimen [12, 24]. Assuming 95% confidence level (first type alpha error 5%) and 90% power, the sample size was calculated as 19 cases in each group. After accounting for a 10% dropout rate, the minimum sample size should be 22 patients in each group. Categorical variables were presented as frequencies and proportions (%) and assessed with Chi-square test or Fisher's exact test. Continuous variables were described as mean ± SD or median with inter-quartile range (IQR). Independent t-test was used for comparison between groups when the data were normally distributed; otherwise, the Mann–Whitney test was used. P values were adjusted with Bonferroni correction for multiple testing. Associations of EFR with target concentration or AUC_24_/MIC were assessed by Spearman correlation coefficient test. The correlation would be interpreted as follows: ${\mid }r{\mid }\, > \,0.70$ is considered strong correlation; $0.50\, < \,{\mid }r{\mid }\, < \,0.70$, moderately strong correlation; $0.3\, < \,{\mid }r{\mid }\, < \,0.5$ , weak to moderately strong correlation; ${\mid }r{\mid }\, < \,0.3$, weak correlation. Statistical analysis was performed using SPSS 22.0 (SPSS, Inc., USA) and the GraphPad Prism 9.0 (GraphPad, Inc., USA) software, and differences with P < 0.05 were considered statistically significant.

## Results

### Demographic and clinical characteristics

**Fig. 1:**
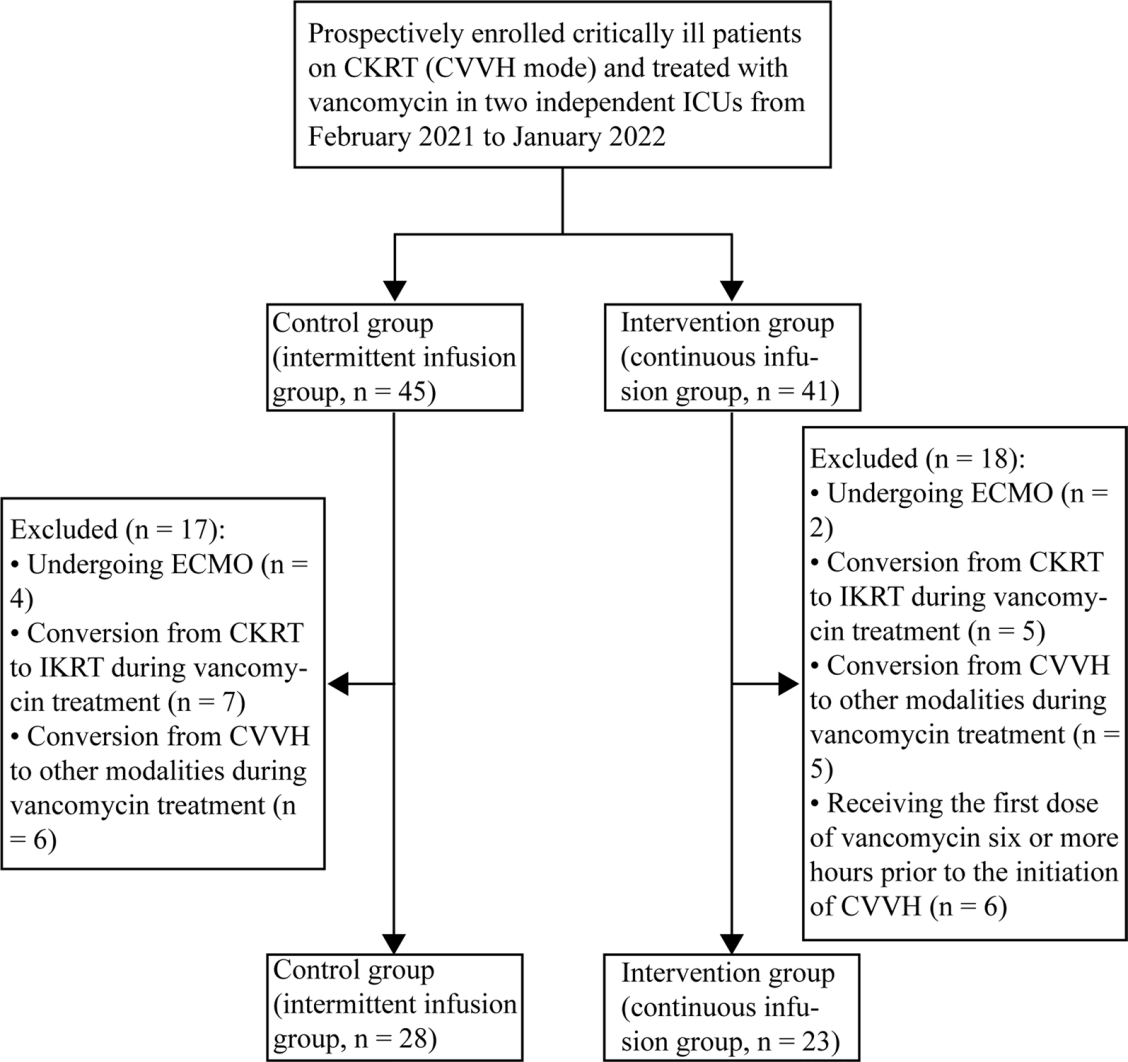
Flow chart of participant selection. Study population selection and criteria for exclusion, a total of 51 patients were included in the analysis. CKRT, continuous kidney replacement therapy; CVVH, continuous venovenous hemofiltration; ICUs, intensive care units; ECMO, extracorporeal membrane oxygenation; IKRT, intermittent kidney replacement treatment

A total of 51 patients met the criteria were enrolled in the study (Fig. 1), 28 of whom in the II group and 23 in the CI group. As shown in Table 1, no significant differences in demographic and clinical characteristics were found between the two groups.

**Table 1:** Demographic and clinical characteristics of the study population

Characteristics	II group (n = 28)	CI group (n = 23)	Statistics	*P*
Demographics				
Gender (male), n (%)	20 (71.4)	16 (69.6)	0.021	0.884
Age (y), mean ± SD	70.2 ± 17.1	72.1 ± 17.1	0.634	0.529
Body weight (kg), mean ± SD	59.8 ± 11.1	61.6 ± 7.0	0.722	0.474
Height (cm), median [IQR]	170.0 (157.0,174.0)	170.0 (161.5, 172.3)	− 0.162	0.872
Assessment scores				
APACHE II	24.8 ± 8.8	22.8 ± 6.0	0.443	0.666
SOFA	10.7 ± 3.5	9.3 ± 2.5	0.694	0.508
Comorbidities, n (%)				
Cerebral infarction	8 (28.6)	5 (21.7)	0.310	0.577
Craniocerebral injury	7 (25.0)	3 (13.0)	0.512	0.474
Hypertension	15 (53.6)	12 (52.2)	0.010	0.921
Diabetes	9 (32.1)	7 (30.4)	0.017	0.896
Congestive heart failure	11 (39.3)	8 (34.8)	0.110	0.741
Atrial fibrillation	4 (14.3)	6 (26.1)	0.493	0.483
COPD	1 (3.6)	2 (8.7)	NA	0.583
Complications, n (%)				
Respiratory failure	22 (78.6)	17 (73.9)	0.152	0.696
Septic shock	20 (71.4)	14 (60.9)	0.634	0.426
Laboratory values at baseline				
Albumin (g/L), mean ± SD	30.9 ± 5.3	32.8 ± 3.4	1.659	0.101
ALT (U/L), median [IQR]	25.0 (16.5, 44.3)	24.0 (18.8, 40.8)	− 0.191	0.848
Creatinine (μmol/L), median [IQR]	153.8 (79.6, 313.0)	151.7 (50.8, 197.0)	− 0.858	0.391
Laboratory values on the last day of vancomycin administration				
Albumin (g/L), median [IQR]	32.4 (26.7, 37.0)	33.1 (30.0, 36.0)	− 1.015	0.310
ALT (U/L), median [IQR]	21.0 (14.0, 34.3)	26.5 (15.5, 39.0)	− 0.910	0.363
Creatinine (μmol/L), median [IQR]	122.5 (64.8, 283.0)	128.1 (79.6, 196.1)	− 0.288	0.774
Dosage regimen				
Daily dose (mg/kg), median [IQR]	21.4 (12.0, 29.6)	16.7 (14.2,26.5)	− 0.395	0.693
Duration (d), median [IQR]	10.0 (8.0, 12.0)	8.0 (6.8, 14.0)	− 0.819	0.413
Concomitant antibiotic therapy, n (%)	20 (71.4)	16 (69.6)	0.021	0.884
Carbapenems	12 (60.0)	11 (68.8)	0.731	0.882
β-Lactamase inhibitor compound	5 (25.0)	4 (25.0)		
Ceftazidime	3 (15.0)	1 (6.3)		
Antifungal agents	3 (15.0)	3 (18.8)	NA	> 0.999
Sites of infection, n (%)				
Lung	26 (92.9)	20 (87.0)	0.497	0.481
Bloodstream	3 (10.7)	3 (13.0)	NA	> 0.999
Urinary tract	4 (14.3)	1 (4.4)	0.51	0.475
Intra-abdominal	1 (3.6)	NA	NA	> 0.999
Skin and soft-tissue	4 (14.3)	NA	NA	> 0.999
Infective endocarditis	1 (3.6)	2 (8.7)	0.032	0.857
≥ 2 sites of infection	14 (50.0)	6 (26.1)	3.029	0.082
Microbiology, n (%)				
MRSA	9 (50.0)	11 (61.1)	2.115	0.861
*Enterococcus faecium*	5 (27.8)	4 (22.2)		
*Staphylococcus epidermidis*	1 (5.6)	2 (11.1)		
*Staphylococcus haemolyticus*	2 (11.1)	1 (5.6)		
*Staphylococcus hominis*	1 (5.6)	NA		
≥ 2 kinds of Gram-positive bacteria	2 (7.1)	NA	NA	0.495
Gram-negative bacteria, n (%)	19 (67.9)	14 (60.9)	0.270	0.603
Fungus	3 (10.7)	3 (13.0)	NA	> 0.999
Vancomycin MIC values, n (%)				
≤ 1 mg/L	13 (81.3)	16 (88.9)	NA	0.648
2 mg/L	3 (18.8)	2 (11.1)	NA	0.648

*II group* intermittent infusion group; *CI group* continuous infusion group; *SD* standard deviation; *IQR* interquartile range; *APACHE II* acute physiology and chronic health evaluation II; *SOFA* sequential organ failure assessment; *COPD* chronic obstructive pulmonary disease; *ALT* alanine aminotransferase; *MRSA* methicillin-resistant *Staphylococcus aureus*; *MIC* minimum inhibitory concentration; *NA* not applicable

### CKRT parameters

Comparison of the CKRT settings for both groups is shown in Table 2. Median EFR was 37.4 (IQR 33.8 to 44.7) mL/kg/h for the II group and 35.6 (IQR 32.8 to 39.9) mL/kg/h for the CI group, respectively. The EFR applied for patients in the two groups all exceeded 25 mL/kg/h. The total clearance and CVVH clearance of vancomycin in the II group was comparable to that in the CI group (3.42 ± 0.78 L/h vs. 3.23 ± 1.08 L/h, P = 0.343; 2.85 ± 0.38 L/h vs. 2.94 ± 0.31 L/h, P = 0.374). The proportion of CVVH clearance to total clearance was 80.3 ± 18.4% and 89.0 ± 11.3%.

**Table 2:** CKRT parameters within 24 h before measuring target concentration in the two groups

CKRT parameters	II group (n = 28)	CI group (n = 23)	Statistics	*P*
Hematocrit (%), median [IQR]	23.7 (21.0, 27.4)	25.1 (23.2, 29.4)	− 1.570	0.116
Blood flow rate (mL/min), median [IQR]	180.0 (142.5, 180.0)	180.0 (150.0, 180.0)	− 0.909	0.363
Pre-dilution replacement fluid rate (mL/h), median [IQR]	1200.0 (1000.0, 1525.0)	1100.0 (1000.0, 1500.0)	− 1.005	0.315
Post-dilution replacement fluid rate (mL/h), median [IQR]	900.0 (800.0, 1050.0)	1000.0 (900.0, 1050.0)	− 0.836	0.403
Net removal fluid rate (mL/h), median [IQR]	194.0 (107.5, 296.3)	200.0 (100.0, 239.0)	− 0.025	0.980
Input of fluids (mL), median [IQR]	2700.5 (1877.5, 3919.8)	2619.0 (2298.8, 3488.0)	− 0.008	0.993
Output of fluids (mL), median [IQR]	3108.0 (2248.5, 3865.5)	2777.5 (2121.8, 3291.8)	− 1.133	0.257
EFR (mL/kg/h), median [IQR]	37.44 (33.8, 44.7)	35.6 (32.8, 39.9)	− 1.233	0.218
Haemofiltration rate (mL/h), median [IQR]	2290.3 (2038.1, 2763.8)	2161.5 (1937.6, 2588.1)	− 1.159	0.247
S_C_, mean ± SD	0.65 ± 0.05	0.67 ± 0.04	− 1.007	0.318
CL_CVVH_ (L/h), mean ± SD	2.85 ± 0.38	2.94 ± 0.31	− 0.896	0.374
CL_total_ (L/h), mean ± SD	3.42 ± 0.78	3.23 ± 1.08	0.954	0.343

*II group* intermittent infusion group; *CI group* continuous infusion group; *IQR* interquartile range; *EFR* effluent flow rate; *SC* sieving coefficient; *CL*_*CVVH*_ clearance of vancomycin in CVVH mode; *CL*_*total*_ clearance of vancomycin

### Distributions of target concentration and AUC_24_/MIC

**Fig. 2:**
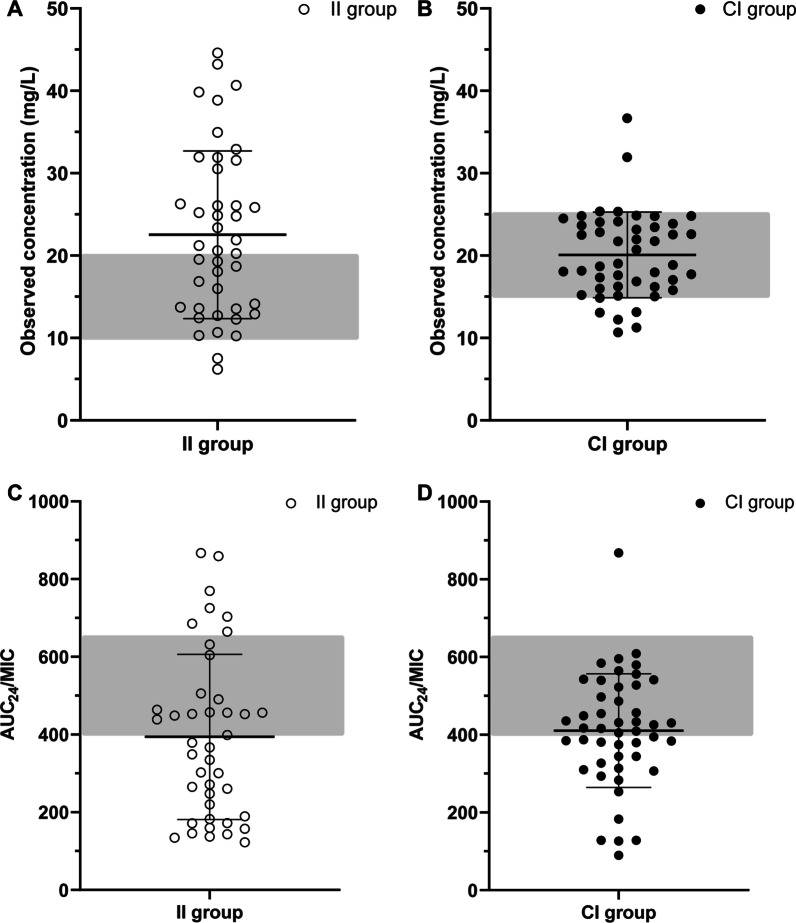
Scatter plot of observed vancomycin concentration in the II group (**A**) and CI group (**B**). Scatter plot of AUC_24_/MIC in the II group (**C**) and CI group (**D**). Solid line represents the mean ± SD. The gray area represents the target range

Distributions of target concentration and AUC_24_/MIC in the II and CI group are illustrated in Fig. 2. The initial mean C_min_ was 23.4 ± 10.0 mg/L for the II group, whereas in the CI group, mean C_ss_ was 19.7 ± 6.5 mg/L. The initial AUC_24_/MIC ratio was 449.3 ± 228.0 for patients treated with II regimen and 414.9 ± 152.2 for patients receiving CI of vancomycin. Increasing and decreasing the daily dose of vancomycin was recommended for 2 patients and 3 patients in the CI group, respectively While in the II group, the daily vancomycin dose was increased in 1 patient and decreased in 6 patients. With the dose adjustment, overall mean C_min_ and C_ss_ were 22.5 ± 10.2 mg/L and 20.1 ± 5.2 mg/L for the II group and CI group. The overall AUC_24_/MIC in patients receiving II and CI of vancomycin was 393.9 ± 202.3 and 410.5 ± 146.4, respectively. Furthermore, higher variability in vancomycin serum concentrations (RSD 44.3% vs. 25.9%) and AUC_24_/MIC (RSD 51.4% vs. 35.7%) were observed in patients treated with II regimen compared with those receiving CI.

### Target attainment of target concentration and AUC_24_/MIC

A total of 89 vancomycin concentrations were available in the final analysis, 42 from the II group and 47 from the CI group. For the II group, initial concentration was subtherapeutic for 3.6% of patients (1/28), supratherapeutic in 64.3% of patients (18/28) and therapeutic in 32.1% of patients (9/28). Whereas in the CI group, initial subtherapeutic concentrations were found in 5 patients (21.7%), therapeutic concentrations were found in 15 patients (65.2%) and supratherapeutic concentrations were found in 3 patients (13.0%). Among overall concentrations were measured in the patients of II group, 2 out of 42 (4.8%) were subtherapeutic, 17 out of 42 (40.5%) were therapeutic and 23 out of 42 (54.8%) were supratherapeutic. Whereas in the CI group, the corresponding ratio was 12.8% (6/47), 78.7% (37/47), 8.5% (4/47), respectively.

The proportion of initial or total concentrations fell into subtherapeutic, therapeutic, and supratherapeutic range in the CI group was significant different from that in the II group (P < 0.001; P < 0.001). Multiple comparison showed that significantly more initial subtherapeutic, therapeutic concentration and less supratherapeutic concentrations were achieved in the CI group (all adjusted P < 0.05). Regarding overall concentrations, significant differences in the percentage of therapeutic, and supratherapeutic levels were found between the two groups (both adjusted P < 0.05).

**Fig. 3:**
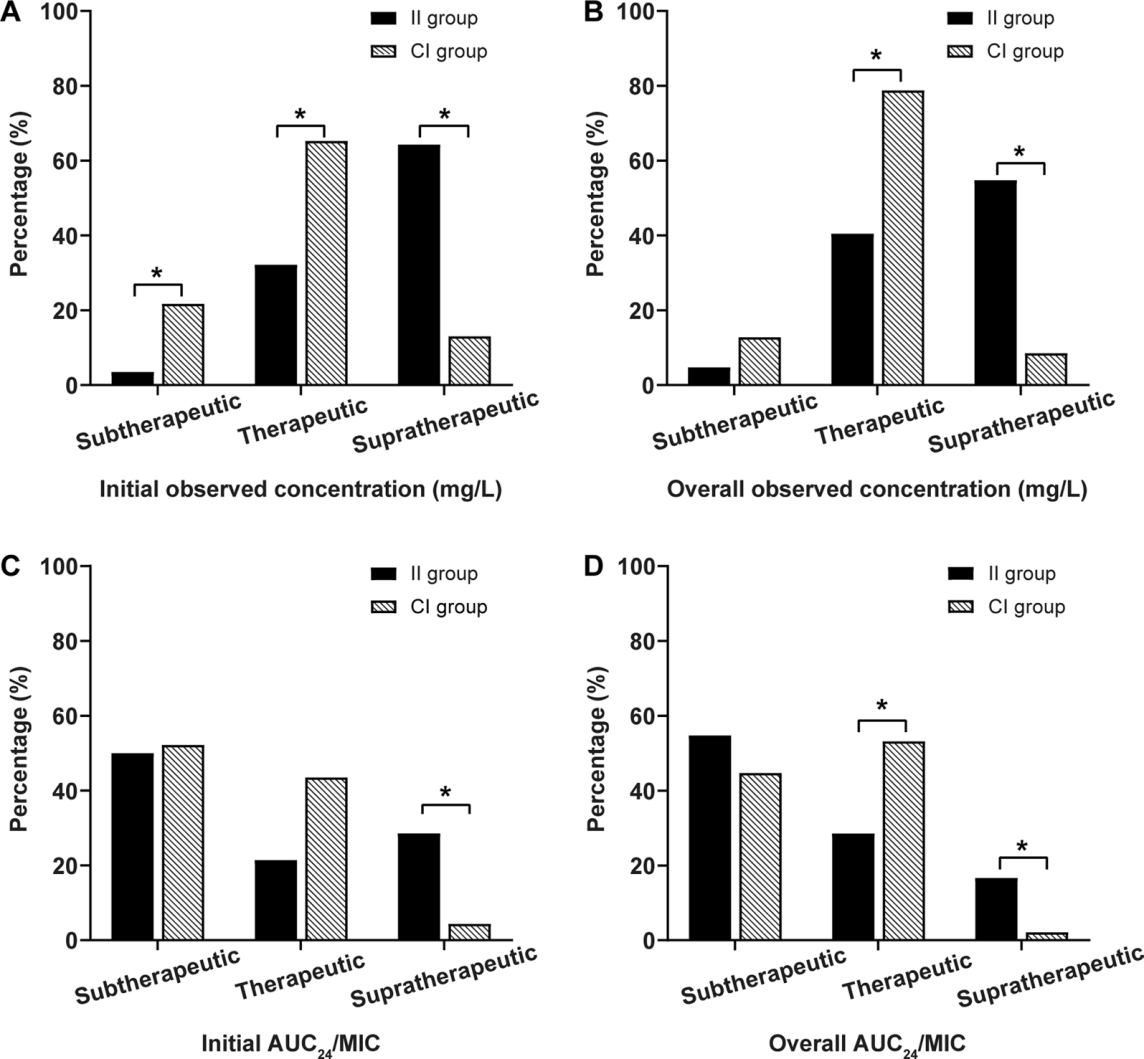
Target attainment of initial observed concentration (**A**) and overall observed concentration (**B**) in the II group and CI group. Target attainment of initial AUC_24_/MIC (**C**) and overall AUC_24_/MIC (**D**) in the II group and CI group during CVVH^a^. ^a^For target concentration, therapeutic exposure is defined as trough concentration of 15–25 mg/L for continuous infusion (CI group) and steady-state concentration of 10-20 mg/L for intermittent infusion group (II group), respectively. For AUC_24_/MIC target, therapeutic exposure is defined as 400–650 for both groups. Suptherapeutic exposure is defined as the target PK/PD indices above the desired range, whereas subtherapeutic exposure is defined as PK/PD indices below the desired range. *Bonferroni-adjusted P < 0.05

The proportion of initial or overall AUC_24_/MIC fell in to the three different ranges between the two groups were significant different (P = 0.040; P = 0.011). Compared with the II group, target attainment of overall AUC_24_/MIC in the CI group was higher (53.2% vs. 28.6%; adjusted P < 0.05). On the other hand, the percentage of AUC_24_/MIC reaching supratherapeutic range was significant higher in the II group when compared with CI group, irrespective of initial AUC_24_/MIC (28.6% vs. 4.4%; adjusted P < 0.05) or overall AUC_24_/MIC (16.7% vs. 2.1%; adjusted P < 0.05). The results are shown in Fig. 3.

### Assessments of therapeutic efficacy and safety

Although a higher rate of clinical success was observed in the CI group when compared to the II group, the difference was not statistically significant (72.2% vs. 50.0%, P = 0.183). Bacterial eradication rates were similar between the two groups (83.3% vs. 75.0%, P = 0.681). One patient in the CI group developed leukopenia, none of the patients in the II group experienced any adverse effects. Mortality between the two groups was similar (21.7% vs. 17.9%, P = 0.728). The results are displayed in Table 3.

**Table 3:** The therapeutic efficacy and safety evaluation of vancomycin administrated in critically ill patients

Items	II group (n = 28)	CI group (n = 23)	Statistics	*P*
Targeted antimicrobial therapy, n (%)	16 (57.1)	18 (78.3)	2.534	0.111
Clinical success, n (%)	8 (50.0)	13 (72.2)	1.771	0.183
G^+^ bacterial eradication, n (%)	12 (75.0)	15 (83.3)	NA	0.681
Pesumed eradication	1 (8.3)	2 (13.3)	NA	> 0.999
Documented eradication	11 (91.7)	13 (86.7)		
Adverse reactions, n (%)				
Leukopenia	0 (0.0)	1 (4.4)	NA	0.462
28-day mortality, n (%)	5 (17.9)	5 (21.7)	0.121	0.728

*II group* intermittent infusion group; *CI group* continuous infusion group; *NA* not applicable

### Correlation results

**Fig. 4:**
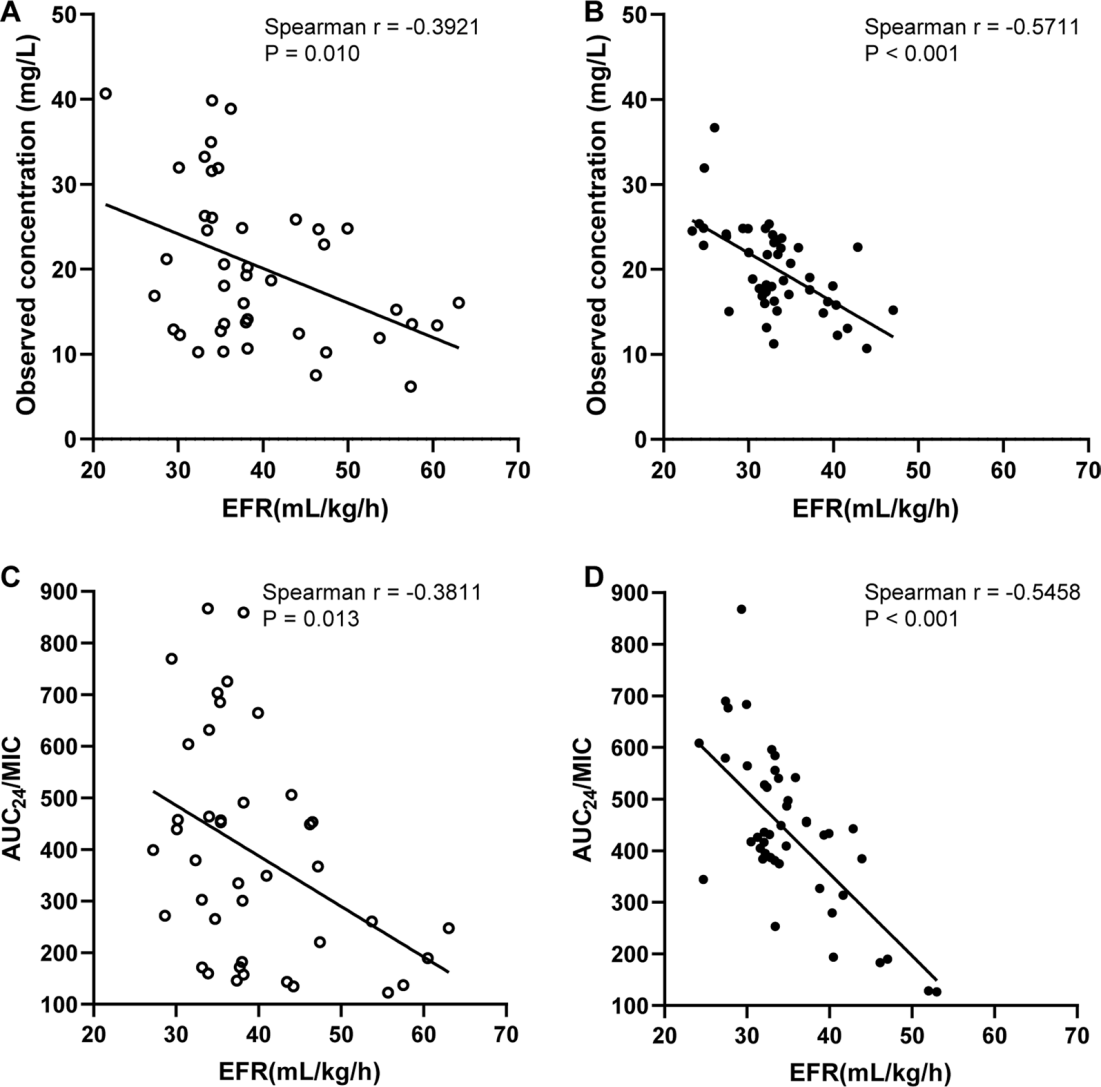
Correlation analysis of target PK/PD indices with EFR. Correlation of observed concentration with EFR in the II group (**A**) and CI group (**B**). Correlation of AUC_24_/MIC with EFR in the II group (**C**) and CI group (**D**). The Spearman correlation coefficient r is shown. Statistical significance was assessed by Spearman correlation. EFR, effluent flow rate

EFR was weak to moderately negatively correlated with observed concentration (r = − 0.3921, P = 0.01), AUC_24_/MIC (r = − 0.3811, P = 0.013) in the II group, whereas this correlation was stronger between EFR and observed concentration (r = − 0.5711, P < 0.001) or AUC_24_/MIC (r = − 0.5458, P < 0.001) in the CI group. The correlation results are presented in Fig. 4.

## Discussion

To our knowledge, this is the first prospective interventional study to compare the target attainment of vancomycin administration, efficacy and safety between II and CI regimen. This prospective interventional study identified that CI of vancomycin was associated with greater target attainment, including target concentration and AUC_24_/MIC, although no significant difference in efficacy and safety outcomes between the two dosing modalities were found. Furthermore, the negative correlation between PK/PD indices and EFR was stronger in patients administrated with vancomycin via CI strategy, which may be partly explained by the higher proportion of CVVH clearance to total clearance in the CI group than in the II group. The proportion of CVVH clearance to total clearance was both higher than 80% in two groups, higher than literature study [20]. This result maybe related to our patients enrolled had anuria during CVVH. Most AKI patients have no urine output, therefore the renal clearance of these patients was few.

Of note, a higher variability in vancomycin concentration was observed in critically ill patients treated with intermittent dosing during CVVH, which was consistent with previous research [25]. The differences in exposure variability between the two modalities might be largely explained by the variability of pharmacokinetic parameters of vancomycin, such as a high variation in volume of distribution and clearance for patients treated with II regimen and change of clearance for patients received with CI [24]. In addition, all critically ill patients receiving II regimen underwent CVVH with EFR exceeding 25 mL/kg/h, the dose and dosing frequency were increased based on recommendations of guideline, thus increasing the variability of target PK/PD parameters.

Similar to that found in Lin's study [12], the present study demonstrated that a higher proportion of target concentration was achieved when vancomycin administrated through CI than II strategy. It should be noted that a direct comparison of AUC_24_/MIC target attainment between the two modalities employed in critically patients during CVVH was lacking in the previous research. Based on clinical efficacy and safety data, trough-only monitoring of vancomycin in patients with serious infections is no longer recommended in the updated IDSA 2020 guideline [13]. Several studies [26–28] have suggested that AUC-guided vancomycin dosing was associated with a significantly reduced risk of nephrotoxicity compared with trough-guided dosing. Therefore, AUC-guided dosing was proposed as an alternative optimal approach to manage vancomycin dosing [13]. Our study showed that target AUC_24_/MIC ratio was more frequently attained in patients during CVVH who received CI than those receiving II, which was similar to that found in prior studies conducted in critically ill patients [24, 29]. However, patients who underwent CKRT were excluded in the prior studies, which was different from the special population enrolled in our study.

Lin et al. [12] found that II of vancomycin during CVVH achieved a higher proportion of initial subtherapeutic serum level than CI regimen. On the contrary, another study [10] showed that more patients receiving CI of vancomycin during CVVH attained concentrations in the subtherapeutic range compared with those treated with II regimen, which was in line with our result. The discrepancy observed between the studies may be attributed to the fact that dosage regimens varied across the studies as well as the definition of target ranges. Our study showed that the percentage of PK/PD parameters fell into supratherapeutic range was significant higher in patients administered via II when compared with those via CI. As reported by Akers et al. [11] patients in the II group receiving high intensity of CVVH was associated with more frequent supratherapeutic levels compared to CI, which was consistent with our result. Nevertheless, a direct comparison of AUC_24_/MIC achieving supratherapeutic range between CI and II regimens was lacking in the prior study. This may be explained by that higher initial dosage or dosing frequency of vancomycin was applied in patients treated with II strategy concomitantly undergoing CVVH with high EFR in order to reduce underexposure of vancomycin. After dose adjustment, the proportion of overall PK/PD indices attained the supratherapeutic range was still greater in the II group. It suggested that accumulation of vancomycin may occur in the patients receiving II regimen and the exposure may not significantly decrease even though reduction of dosage. Therefore, CI of vancomycin is a preferred approach under the circumstances.

Despite target attainment was achieved greater in patients treated with CI compared with those on II in critically ill adults during CVVH, no significant difference in therapeutic outcomes was found, which was in agreement with a previous meta-analysis [30] and a retrospective multicentre matched cohort study [31]. The therapeutic efficacy was affected by many factors, such as severity of infection, site of infection, the presence of co-morbidities, concomitant Gram-negative or fungal infections, etc. The non-significant result may also be attributed to the small sample size of the study, which was underpowered to observe a difference between groups.

A retrospective cohort study [32] of critically ill adults administrated with vancomycin on concurrent CVVH suggested that an inverse association existed between EFR and vancomycin trough concentrations. However, there was a lack of research on the correlation between EFR and AUC_24_/MIC in the previous study. Our study demonstrated that EFR was associated with AUC_24_/MIC as well as target concentration of vancomycin in critically ill patients who underwent CVVH. Moreover, the magnitude of the correlation was also compared in our research, which indicated that CI dosing regimen could be designed based on the intensity of CVVH, since the negative relationship between EFR and PK/PD parameters was stronger in the patients employing CI regimen.

Compared with II, CI has many advantages. First of all, CI of vancomycin could prolong the time that the antibiotic concentration is maintained above the MIC since its antibacterial activity is time dependent [33]. Moreover, a more accurate estimation of AUC_24_ appears to be achieved via CI, given that a constant C_ss_ is maintained using this approach, which could eliminate the peak-trough variability with use of II [34]. In addition, CI of vancomycin simplifies TDM through a single concentration measured at any time after steady state is reached [35]. Whereas a trough level for the II should be measured after achievement of steady-state concentration and within 30 min prior to the administration of the next dose of vancomycin. Furthermore, in a meta-analysis of cohort studies and randomized controlled trials, CI of vancomycin was associated with greater attainment of therapeutic concentration [36]. On the other hand, several studies found that CI of vancomycin provided higher target attainment of AUC_24_/MIC in comparison with II regimen [24, 29, 37]. Lastly, recent reviews have indicated CI is associated with reduced cost in comparison with II [38].

Several limitations of this investigation should be considered when interpreting our findings. First, the major limitation was that this was not a randomized study. There may be had bias in the evaluation of treatment outcome in CI and II regimens of vamcomycin. We were unable to clearly demonstrate the clinical superiority of CVI as compared with IVI in patients with CRRT. Second, this study was limited by a small sample size, which may not detect the significant differences regarding safety and efficacy outcomes. Third, despite a higher target attainment of PK/PD parameters, no significant difference in therapeutic efficacy was found between the CI and II regimens. Other potentially confounding factors, including concomitant Gram-negative, fungal infections and co-morbidities, also likely have an impact on the assessment of therapeutic efficacy. Fourth, since all critically ill patients underwent CVVH with EFR exceeding 25 mL/kg/h in the II group, increased dose and frequency of vancomycin was administrated on the basis of guideline recommendation, given that the exposure of vancomycin might not be adequate. Consequently, the possibility of bias might be increased. Fifth, vancomycin clearance was only estimated based on fraction originated from CKRT, non-kidney clearance of vancomycin was not included in our study. Lastly, the modalities of CKRT used exclusively in our study was CVVH, the possibility of generalizing our results to other CKRT modes including continuous venovenous haemodialysis or continuous venovenous haemodiafiltration was limited, as clearance of vancomycin varied among different modalities of CKRT. A prospective, randomized controlled trial with a larger sample size comparing CI vs. II in critically ill patients receiving vancomycin during CKRT in the future should be performed to validate the safety and efficacy as well as pharmacokinetic target attainment.

## Conclusion

Our study suggests that CI of vancomycin in critically ill patients undergoing CVVH is associated with greater PK/PD target attainment, including target concentration and AUC_24_/MIC, as compared with traditional II regimen. Our study also indicates that the inverse correlation of PK/PD indices with EFR was stronger in patients receiving CI of vancomycin.

## Data Availability

The datasets generated and/or analyzed during the current study are not publicly available due to limitations of ethical approval involving the patient data and anonymity but are available from the corresponding author on reasonable request.

## Abbreviations

II: Intermittent infusion
CI: Continuous infusion
CVVH: Continuous venovenous hemofiltration
PK/PD: Pharmacokinetic/pharmacodynamics
AUC_24_/MIC: Area under the curve over 24 h to minimum inhibitory concentration
ICU: Intensive care unit
MRSA: Methicillin-resistant *Staphylococcus aureus*
MRCNS: Methicillin resistant coagulase negative staphylococci
VRE: Vancomycin-resistant enterococci
CKRT: Continuous kidney replacement therapy
TDM: Therapeutic drug monitoring
C_min_: Trough concentration
C_ss_: Steady-state concentration
ECMO: Extracorporeal membrane oxygenation
IRRT: Intermittent kidney replacement treatment
APACHE II: Acute physiology and chronic health evaluation II
SOFA: Sequential organ failure assessment
ALT: Alanine aminotransferase
EFR: Effluent flow rate
IQR: Inter-quartile range
SC: Sieving coefficient
CL_CVVH (post)_: Hemofilter clearance for CVVH using post-filter hemodilution
CL_CVVH (pre)_: Hemofilter clearance for CVVH using post-filter hemodilution
CL_vanc_: Vancomycin clearance
C_SE_: Serum vancomycin concentration in the ultrafiltrate collection period
C_UF_: The concentration of vancomycin in the ultrafiltrate
Q_f_: Haemofiltration rate
Q_b_: Blood flow rate
Q_spre_: Pre-dilution replacement fluid flow rate
